# ﻿On eleven species of jumping spiders from Xishuangbanna, China (Araneae, Salticidae)

**DOI:** 10.3897/zookeys.1116.82858

**Published:** 2022-08-08

**Authors:** Cheng Wang, Shuqiang Li

**Affiliations:** 1 Guizhou Provincial Key Laboratory for Biodiversity Conservation and Utilization in the Fanjing Mountain Region, Tongren University, Tongren, Guizhou 554300, China Tongren University Tongren China; 2 Institute of Zoology, Chinese Academy of Sciences, Beijing 100101, China Institute of Zoology, Chinese Academy of sciences Beijing China

**Keywords:** Morphology, new combination, new genus, new species, Southeast Asia, taxonomy

## Abstract

One new genus and eight new species from Xishuangbanna, China are described and diagnosed: *Bocusoideszhaoi***gen.** and **sp. nov.**, *Euochinmii***sp. nov.** (♂♀), *E.tangi***sp. nov.** (♀), *Eupoalogunovi***sp. nov.** (♂♀), *Indomarengowengnan***sp. nov.** (♀), *Laufeiazhangae***sp. nov.** (♂♀), *Simaethahuigang***sp. nov.** (♂♀), and *Synagelidescheni***sp. nov.** (♀). The unknown sexes of three endemic species, *Chalcovietnamicuslii* (Lei & Peng, 2010) **comb. nov.** (ex *Chalcoscirtus* Bertkau, 1880), *Indomarengoyui* Wang & Li, 2020, and *Rhenetriapophyses* Peng, 1995 are described for the first time.

## ﻿Introduction

As a result of a series of taxonomic studies and biodiversity surveys conducted over the last three decades, knowledge of the salticid fauna from Xishuangbanna, China has increased considerably (Li 2020; Lin and Li 2020; Hong et al. 2022). To date, the list of salticids from Xishuangbanna, including the eight new species described here, comprises at least 145 species, which is more than the number of species in several adjacent countries and regions (Logunov 2021; Wang and Li 2021; WSC 2022). However, there is no doubt that new records and species will be discovered in this biodiversity hotspot and the list of species will be increased further because most area of this region remains insufficiently surveyed (Wang et al 2020; Li et al 2021; Yao et al 2021).

In the current study, eight species collected from Xishuangbanna are recognized as new to science, and the unknown sexes of three endemic species are also described.

## ﻿Materials and methods

Specimens were collected by fogging and sieving leaf litter in the tropical rainforest of Xishuangbanna, China. All specimens are preserved in 75% ethanol and are deposited in the Institute of Zoology, Chinese Academy of Sciences (**IZCAS**) in Beijing, China. Methods follow Wang and Li (2021).

All measurements are given in millimeters. Leg measurements are given as: total length (femur, patella + tibia, metatarsus, tarsus). References to figures in the cited papers are listed in lowercase type (fig. or figs); figures in this paper are noted with an initial capital (Fig. or Figs). Abbreviations used in the text and figures are as follows: **AERW** anterior eye row width; AME anterior median eye; **ALE** anterior lateral eye; **AG** accessory gland; **AR** atrial ridge; **AS** anterior chamber of spermatheca; **At** atrium; **CD** copulatory duct; **CO** copulatory opening; **CP** cymbial process; **DTA** dorsal tibial apophysis; **E** embolus; **ED** embolic disc; **ET** embolic tooth; **EFL** eye field length; **FD** fertilization duct; **H** epigynal hood; **MA** median apophysis; **MP** median plate; **MS** median septum; **PERW** posterior eye row width; **PL** posterior lobe; **PLE** posterior lateral eye; **PS** posterior chamber of spermatheca; **RFA** retrolateral femoral apophysis; **RPA** retrolateral patellar apophysis; **RTA** retrolateral tibial apophysis; **S** spermatheca; **SD** sperm duct; **TA** terminal apophysis; **TF** tibial flange; **TmA** terminal apophysis of embolic division; **VTA** ventral tibial apophysis.

## ﻿Taxonomy

### ﻿Family Salticidae Blackwall, 1841

#### ﻿Genus Bocusoides gen. nov.

Taxon classificationAnimaliaAraneaeSalticidae

DFC614CF-D518-513C-8A29-E791697E948A

https://zoobank.org/4805E7A8-886F-4F82-9134-EF0390496619

##### Type species.

*Bocusoideszhaoi* sp. nov. from China.

##### Etymology.

The generic name is the combination of “oides”, meaning “having the form of”, and the similar genus name *Bocus*; gender masculine.

##### Diagnosis.

**Figure 1.**
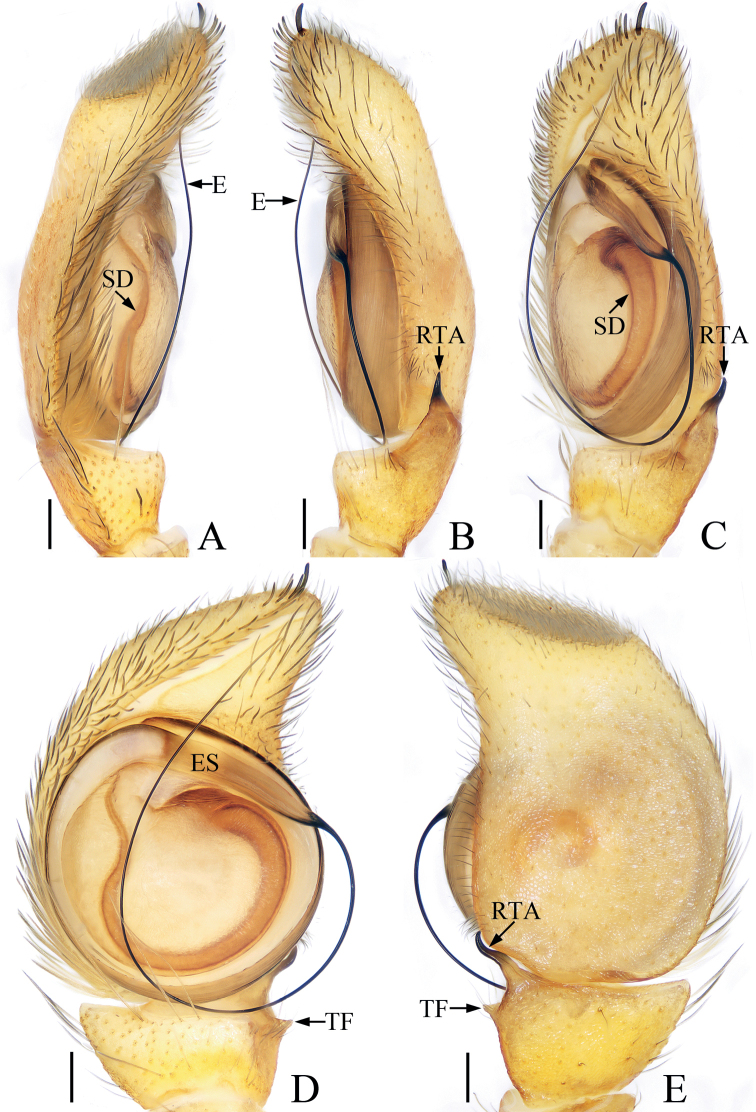
*Bocusoideszhaoi* sp. nov., male holotype palp **A** prolateral **B** retrolateral **C** ventro-lateral **D** ventral **E** dorsal. Scale bars: 0.1.

**Figure 2.**
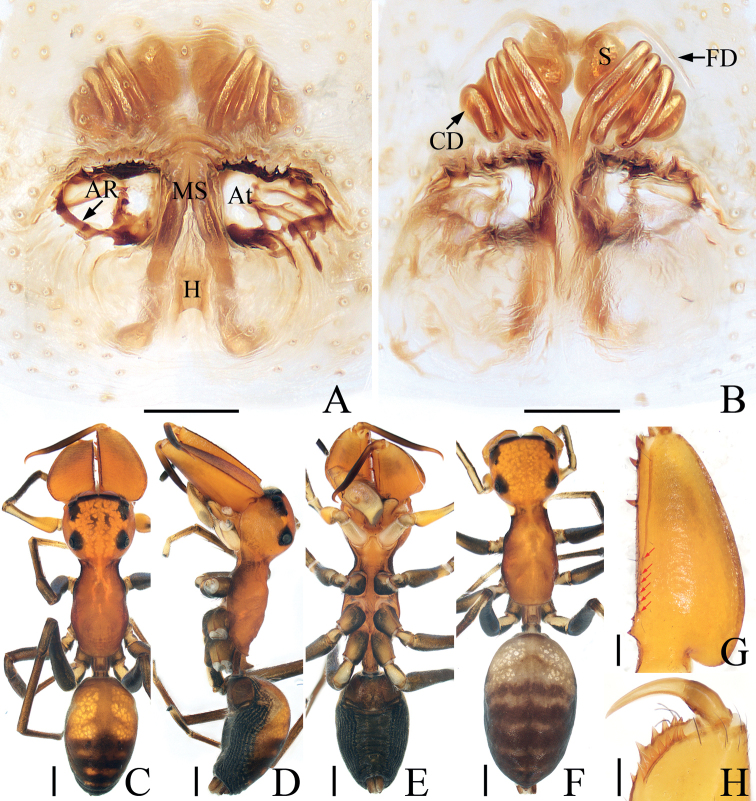
*Bocusoideszhaoi* sp. nov., male holotype and female paratype **A** epigyne, ventral **B** vulva, dorsal **C** male holotype habitus, dorsal **D** ditto, lateral **E** ditto, ventral **F** female paratype habitus, dorsal **G** holotype chelicera, posterior **H** female paratype chelicera, posterior. Scale bars: 0.1 (**A, B, G, H**); 0.5 (**C–F**).

*Bocusoides* gen. nov. can be easily distinguished from other genera of Myrmarachnina, except *Bocus* Peckham & Peckham, 1892, by having an elongated carapace with an obvious postocular constriction and an anteriorly broadened sternum. It can be distinguished from *Bocus* by the following: 1) male cheliceral paturon abruptly broadened at base (Fig. 2C–E, G; Deeleman-Reinhold and Floren 2003: fig. 9) versus gradually broadened from the base to the middle part in *Bocus* (Wanless 1978a: fig. 1C, 2B); 2) carapacal postocular constriction narrower, about 1/2 the carapacal width (Fig. 2C, F; Deeleman-Reinhold and Floren 2003: fig. 9) versus broader, about 2/3 the carapacal width in *Bocus* (Wanless 1978a: figs 1A, 2A); 3) pedical with conical, dorsal process (Fig. 2D; Deeleman-Reinhold and Floren 2003: fig. 11) versus absent in *Bocus* (Wanless 1978a: figs 1H, 2D); 4) leg formula 4123 versus 4132 in *Bocus* (see the description by Wanless 1978a); 5) male abdomen not constricted (Fig. 2C; Deeleman-Reinhold and Floren 2003: fig. 9) versus constricted at anterior 1/3 in *Bocus* (Wanless 1978a: figs 1A, 2A); 6) male palp with short tibia, which is wider than long and with filiform embolus with proximal disc (Fig. 1D; Deeleman-Reinhold and Floren 2003: fig. 13) versus tibia longer, at least as long as wide, and flat embolus without proximal disc in *Bocus* (Wanless 1978a: fig. 3A, E); 7) epigyne with distally circled copulatory ducts, elongated spermathecae, and elongated, arched fertilization ducts (Fig. 2A, B; Deeleman-Reinhold and Floren 2003: fig. 17) versus copulatory ducts not circled, spermathecae spherical, and fertilization ducts ordinary in *Bocus* (Wanless 1978a: fig. 1G).

##### Description.

Medium-sized, ant-like spiders. Carapace elongated. Pedicel short, with dorsal, conical process. Chelicerae well developed and with paturon abruptly broadened at base in males. Endites longer than wide, distally bearing dense, dark setae. Labium slightly darker than endites. Sternum irregular, anteriorly broadened. Legs elongated, with 11 and five ventral spines on tibiae and metatarsi I, respectively. Abdomen oval, not constricted; dorsum with white guanine patches or yellow-sliver spots at the lateral sides of anterior half, and alternate dark and paler transverse bands posteriorly, entirely covered by scutum in males; venter dark-brown.

Palp: tibia wider than long, with tapered, short retrolateral apophysis and triangular flange; cymbium flat, setose, with an apical spine; bulb flat and almost round, with tapered sperm duct extending along the submargin; embolus twice coiled, the first forming a broad, flat circle, the second with an elongated, lamellar disc followed by filiform remainder.

Epigyne: with posteriorly located hood; atria paired, oval, medially located, with arched lateral ridges; copulatory openings hidden; copulatory ducts membranous at origin, followed by sclerotized portion ascending obliquely, distally coiled four circles, which are encircling or lateral to the elongated spermathecae; fertilization ducts slender, arched, originating from the anterior portions of spermathecae.

##### Composition.

The genus currently includes the type species and *B.angusticollis* (Deeleman-Reinhold & Floren, 2003) comb. nov.

#### ﻿ Bocusoides zhaoi sp. nov.

Taxon classificationAnimaliaAraneaeSalticidae

4A144E64-CDE6-5042-8009-EA1A68A84140

https://zoobank.org/C17F2855-F271-40D6-9137-DDDC0C2F1E0F

Figs 1 , 2

##### Type material.

***Holotype*** ♂ (IZCAS-Ar42904), China: Yunnan: Xishuangbanna, Mengla County, Menglun Town, Xishuangbanna National Nature Reserve, 200 m east of Lvshilin, artificial forest (21°57.95'N, 101°12.30'E, ca 780 m alt.), 13.viii.2011, Q. Zhao leg. ***Paratypes*** 2♂7♀ (IZCAS-Ar42905–42913), same data as holotype; 1♂2♀ (IZCAS-Ar42914–42916), 55 km from Xishuangbanna National Nature Reserve, secondary forest (21°57.99'N, 101°12.17'E, ca 840 m alt.), 18.viii.2011, Q. Zhao leg.

##### Etymology.

The specific name is a patronym in honor of Qingyuan Zhao, the collector of this new species; noun (name) in genitive case.

##### Diagnosis.

*Bocusoideszhaoi* sp. nov. closely resembles *B.angusticollis* comb. nov. from Borneo in having a similar habitus and copulatory organs, but it can be easily distinguished by the following: 1) width of embolic disc greater than largest diameter of visible sperm duct (Fig. 1D) versus less than 1/2 in *B.angusticollis* (Deeleman-Reinhold and Floren 2003: fig. 13); 2) RTA curved inward distally in ventral view (Fig. 1D) versus curved retrolaterally in *B.angusticollis* (Deeleman-Reinhold and Floren 2003: fig. 13); 3) tibial flange about 1/4 RTA length in dorsal view (Fig. 1E) versus more than 1/2 in *B.angusticollis* (Deeleman-Reinhold and Floren 2003: fig. 14); 4) male chelicerae with three distal promarginal teeth that are almost equal in size, and female chelicerae with six promarginal teeth (Fig. 2G, H) versus male chelicerae include a basal denticle among the three distal promarginal teeth and female chelicerae with only three promarginal teeth in *B.angusticollis* (see the description by Deeleman-Reinhold and Floren 2003); 5) copulatory ducts partially encircle spermathecae (Fig. 2B) versus copulatory ducts lateral to spermathecae in *B.angusticollis* (Deeleman-Reinhold and Floren 2003: fig. 17).

##### Description.

**Male** (Figs 1, 2C–E, G). Total length 5.00. Carapace 2.68 long, 1.23 wide. Abdomen 2.00 long, 1.23 wide. Clypeus 0.03 high. Eye sizes and inter-distances: AME 0.37, ALE 0.19, PLE 0.17, AERW 1.06, PERW 1.06, EFL 0.89. Legs: I 4.88 (1.45, 1.98, 0.90, 0.55), II 4.15 (1.30, 1.60, 0.80, 0.45), III 4.96 (1.55, 1.68, 1.23, 0.50), IV 6.52 (2.13, 2.23, 1.63, 0.53). Carapace elongated, yellow to yellow-brown, covered with dark brown setae at anterior margin, elevated cephalic region and sloped thorax separated by deep constriction. Pedicel short, with dorsal, conical process. Chelicerae broad, with five promarginal and six retromarginal teeth. Endites longer than wide, bearing dense, dark setae distally. Labium slightly darker than endites. Sternum elongated, irregular, about 2.5 times longer than wide. Legs yellow to dark brown, with 11 and five ventral spines on tibiae and metatarsi I, respectively. Abdomen suboval, dorsum with yellow-sliver spots separated by a longitudinal, central, vein-shaped, brown band anteromedially, followed by alternate dark and dark yellow transverse bands, entirely covered by scutum; venter dark brown.

Palp (Fig. 1A–E): tibia wider than long in ventral view, with short, triangular flange, tapered retrolateral apophysis slightly curved into an S-shape at distal half, pointed apically; cymbium flat, setose, with apical bristle; bulb flat, almost round, with tapered sperm duct; embolus twice coiled, the first forming a broad, flat circle, the second with elongated, lamellar disc followed by filiform remainder coiling about 360° and reaching cymbial tip distally.

**Female** (Fig. 2A, B, F, H). Total length 5.59. Carapace 2.68 long, 1.09 wide. Abdomen 2.46 long, 1.50 wide. Clypeus 0.03 high. Eye sizes and inter-distances: AME 0.38, ALE 0.19, PLE 0.17, AERW 1.06, PERW 1.05, EFL 0.83. Legs: I 3.81 (1.18, 1.53, 0.65, 0.45), II 3.33 (1.05, 1.30, 0.60, 0.38), III 4.23 (1.35, 1.43, 1.00, 0.45), IV 5.69 (1.80, 1.98, 1.43, 0.48). Habitus similar to that of male except with less-developed chelicerae with six promarginal and seven retromarginal teeth, a pair of white spots on the lateral margins of carapace constriction, without scutum on dorsum of abdomen.

Epigyne (Fig. 2A, B): slightly longer than wide, with tube-shaped, posteriorly located hood; atria paired, oval, extending transversely, with arched lateral ridges; copulatory openings hidden; copulatory ducts membranous at origin, followed by sclerotized portion ascending obliquely, coiled four times distally; spermathecae elongated, partly encircled by copulatory ducts; fertilization ducts slender, arched, originating from the anterior portions of spermathecae.

##### Distribution.

Known only from the type locality in Yunnan, China.

#### ﻿Genus Chalcovietnamicus

Taxon classificationAnimaliaAraneaeSalticidae

Marusik, 1991

BAFFA92C-E3B8-5D8C-B653-1FDFEDCEA1D7

##### Type species.

*Chalcoscirtusvietnamensis* Żabka, 1985 from Vietnam by subsequent designation.

#### ﻿ Chalcovietnamicus lii

Taxon classificationAnimaliaAraneaeSalticidae

(Lei & Peng, 2010) comb. nov.

A22E1F51-BC90-51F8-852C-48FC6D451874

Figs 3 , 4

Chalcoscirtus lii Lei and Peng 2010: 67, fig. 1A–C (female holotype, not examined).

##### Material examined.

1♂1♀ (IZCAS-Ar42917–42918), China: Yunnan: Xishuangbanna, Mengla County, Menglun Town, Menglun Nature Reserve, Xishuangbanna Tropical Botanical Garden, 1 site in Mafengzhai (21°53.49'N, 101°17.40'E, ca 520 m alt.), 29.iv.2019, C. Wang leg.

##### Diagnosis.

**Figure 3.**
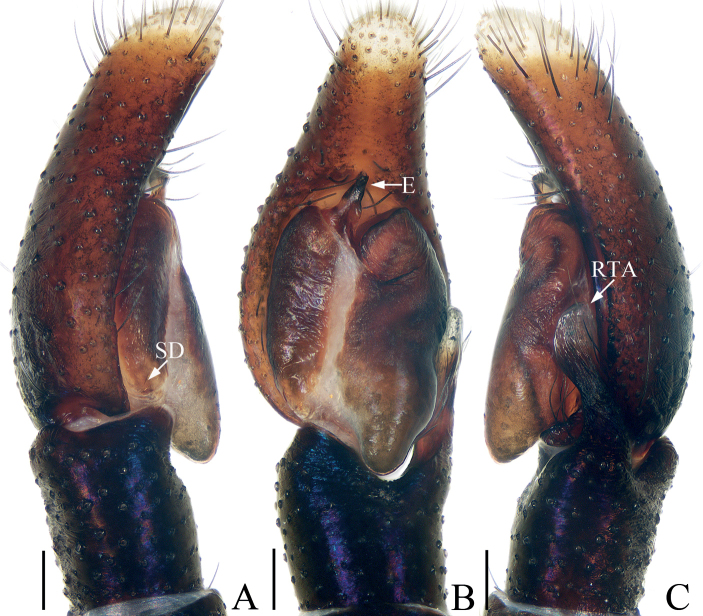
*Chalcovietnamicuslii* comb. nov., male palp **A** prolateral **B** ventral **C** retrolateral. Scale bars: 0.1.

**Figure 4.**
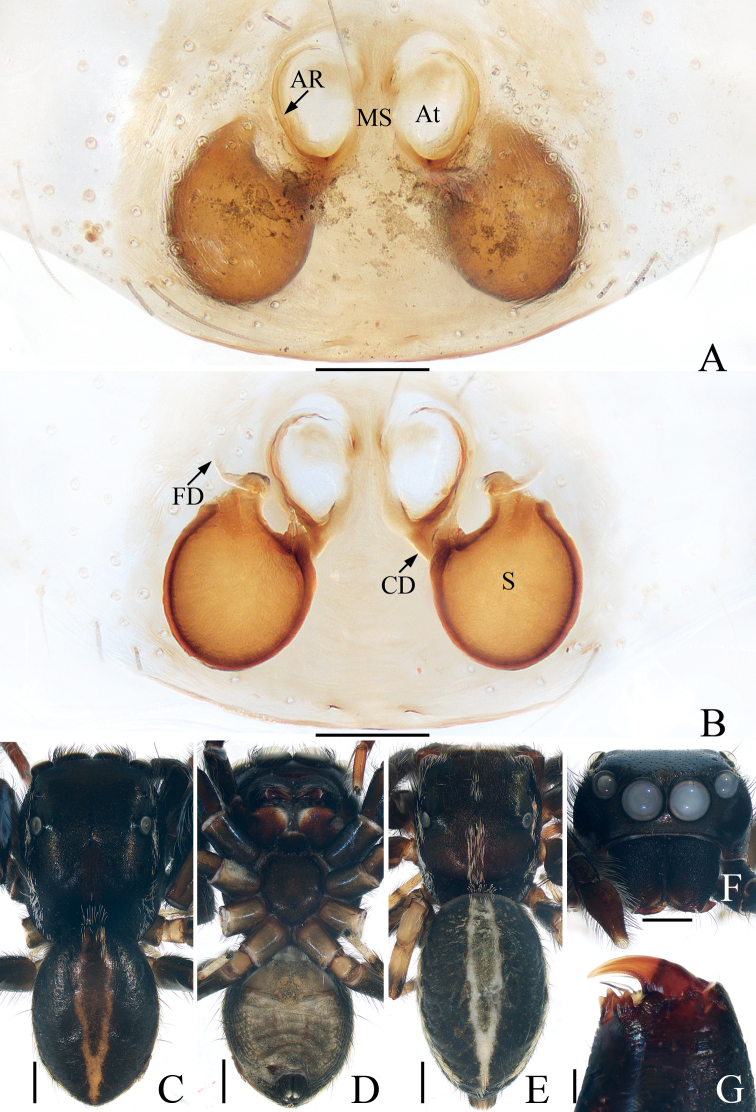
*Chalcovietnamicuslii* comb. nov. **A** epigyne, ventral **B** vulva, dorsal **C** male habitus, dorsal **D** ditto, ventral **E** female habitus, dorsal **F** male carapace, frontal **G** male chelicera, posterior. Scale bars: 0.1 (**A, B, G**); 0.5 (**C–F**).

The male of this species closely resembles that of *C.vietnamensis* (Żabka, 1985) from Vietnam in having a similar palp, but it can be distinguished by the following: 1) embolus with small, semicircular lamellar process (Fig. 3B) versus with a larger, subtriangular process in *C.vietnamensis* (Żabka 1985: fig. 71); 2) RTA broadened and extending anteriorly at distal half (Fig. 3C) versus somewhat tapered, extending antero-prolaterally in *C.vietnamensis* (Żabka 1985: fig. 72). The female of this species resembles that of *Chalcoscirtusparvulus* Marusik, 1991 from Greece, Turkey, Kazakhstan, Iran, and Central Asia in having paired, oval atria and straight, short copulatory ducts, but it can be easily distinguished by the following: 1) spermathecae separated by more than 2/3 their width (Fig. 4A, B) versus touching in *C.parvulus* (Logunov and Marusik 1999: fig. 93); 2) abdomen dorsally with a longitudinal, fusiform stripe across entire surface (Fig. 4E) versus with a pair of bars posteriorly in *C.parvulus* (Logunov and Marusik 1999: fig. 91).

##### Description.

**Male** (Figs 3, 4C, D, F, G). Total length 4.23. Carapace 2.30 long, 1.77 wide. Abdomen 2.24 long, 1.53 wide. Clypeus 0.11 high. Eye sizes and inter-distances: AME 0.49, ALE 0.31, PLE 0.25, AERW 1.50, PERW 1.53, EFL 1.27. Legs: I 6.71 (2.00, 2.95, 1.23, 0.53), II 5.03 (1.53, 2.00, 1.05, 0.45), III 4.21 (1.33, 1.48, 0.95, 0.45), IV 4.46 (1.40, 1.60, 1.01, 0.45). Carapace dark brown, bearing bilateral white and antero-marginal dark setae. Fovea dark, longitudinal, bar-shaped. Chelicerae red-brown to dark, with two promarginal teeth and one retromarginal fissidental tooth with two cusps. Endites paler than chelicerae, with pale ental margins bearing dark, thin setae. Labium dark. Sternum colored as labium, almost heart-shaped, bearing dark setae. Legs I longest, dark brown except tarsi red-brown, with three and two pairs of ventral spines on tibia and metatarsi, respectively; rest of legs yellow, with dark brown femora. Abdomen suboval, dorsum dark brown, with longitudinal, yellow to dark brown fusiform stripe across entire surface, cluster of antero-marginal white setae; venter brown, with pair of rufous, oblique stripes medially, covered with dark, thin setae.

Palp (Fig. 3A–C): tibia thick, slightly longer than wide; retrolateral tibial apophysis narrowed medially, broadened, extending anteriorly at distal half; cymbium almost two times longer than wide, gradually narrowed at distal half in ventral view; bulb suboval, with sperm duct sinuous retrolaterally; embolus short, originating from antero-prolateral portion of bulb, blunt apically, with small, semicircular lamellar process at base.

**Female** (Fig. 4A, B, E). Described by Lei and Peng (2010).

##### Distribution.

Known only from the type locality in Yunnan, China.

#### ﻿Genus Euochin

Taxon classificationAnimaliaAraneaeSalticidae

Prószyński, 2018

1E96056F-A989-59D8-AAB5-754D803C43D5

##### Type species.

*Euophrysatrata* Song & Chai, 1992 from China by original designation.

#### ﻿ Euochin mii sp. nov.

Taxon classificationAnimaliaAraneaeSalticidae

A8AE73B5-3E74-54A4-8E3E-068989A49A16

https://zoobank.org/CEFDA51B-09E0-455C-8454-1FD0423DC48B

Figs 5 , 6

##### Type material.

***Holotype*** ♂ (IZCAS-Ar42919), China: Yunnan: Xishuangbanna, Mengla County, Xiaolongha Village, Xishuangbanna National Nature Reserve, seasonal rainforest (21°24.24'N, 101°36.27'E, ca 710 m alt.), 17.xi.2013, Q. Zhao and Z. Chen leg. ***Paratypes*** 1♂6♀ (IZCAS-Ar42920–42926), same data as holotype; 1♂2♀ (IZCAS-Ar42927–42929), same locality and collectors, 21.xi.2013.

##### Etymology.

The species is named after Prof. Xiaoqi Mi, who helped us greatly with this research; noun (name) in genitive case.

##### Diagnosis.

**Figure 5.**
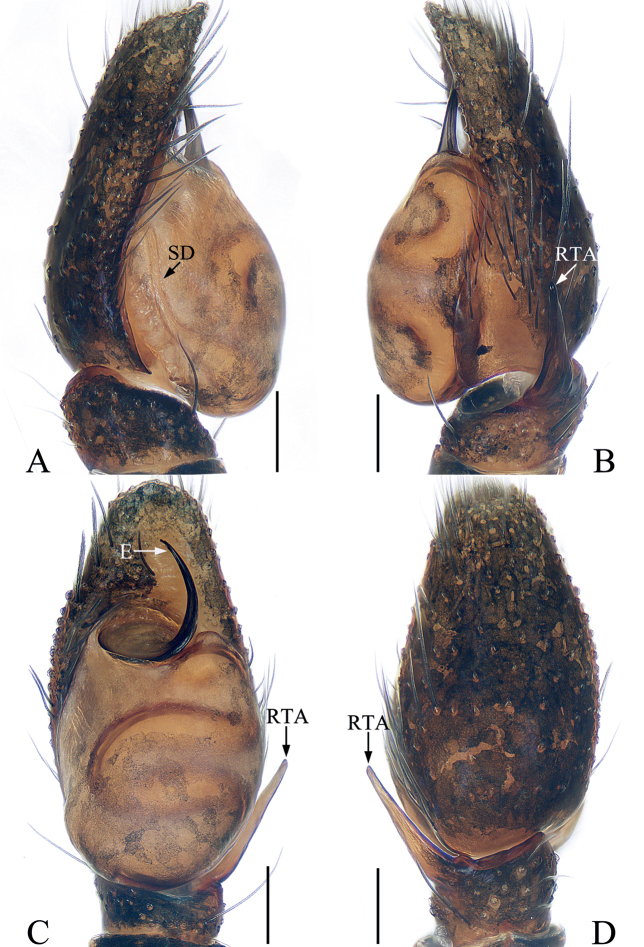
*Euochinmii* sp. nov., male holotype palp **A** prolateral **B** retrolateral **C** ventral **D** dorsal. Scale bars: 0.1.

**Figure 6.**
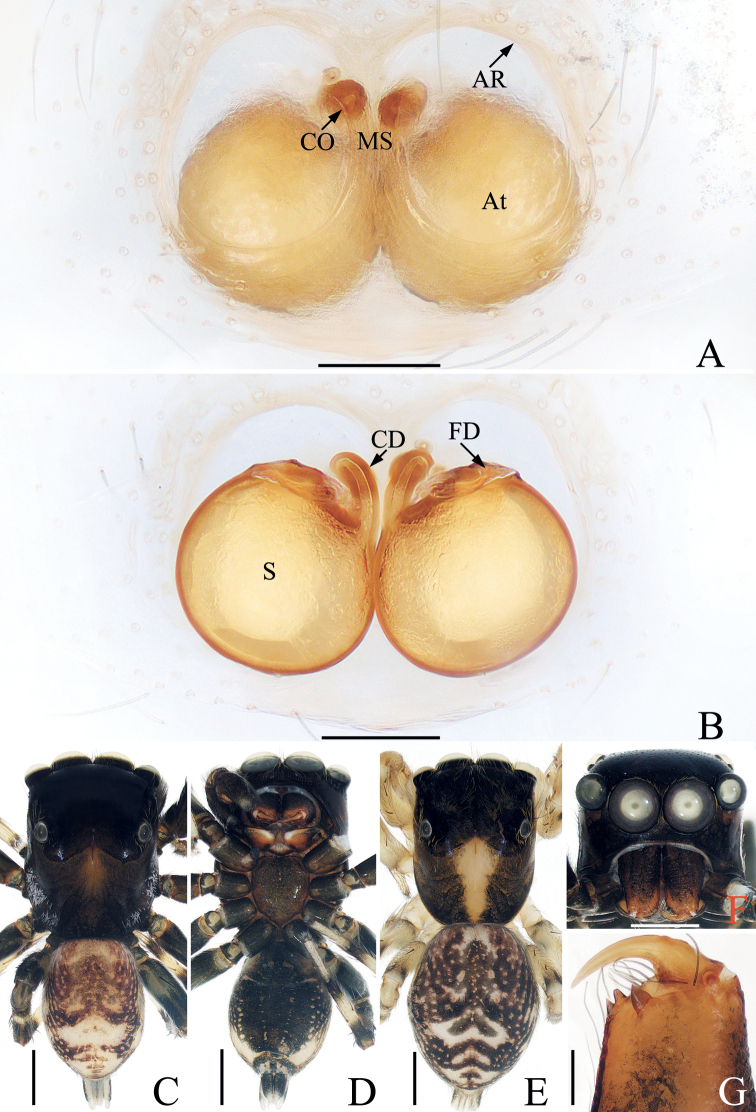
*Euochinmii* sp. nov., male holotype and female paratype **A** epigyne, ventral **B** vulva, dorsal **C** male holotype habitus, dorsal **D** ditto, ventral **E** female paratype habitus, dorsal **F** holotype carapace, frontal **G** holotype chelicera, posterior. Scale bars: 0.1 (**A, B, G**); 0.5 (**C–F**).

*Euochinmii* sp. nov. resembles that of *E.subwanyan* (Wang & Li, 2020) from China in having a tapered embolus, straight retrolateral tibial apophysis and similarly sized, paired atria, but it differs by the following: 1) embolus forming a disc at base (Fig. 5C) versus indistinct in *E.subwanyan* (Wang and Li 2020a: fig. 5C), 2) copulatory ducts about 1/7 diameter of spermatheca and curved anteromedially (Fig. 5C) versus about 1/4 diameter of spermatheca and twisted entirely in *E.subwanyan* (Wang and Li 2020a: fig. 6C). The female also closely resembles *E.luzonica* Logunov, 2020 from Philippines in having thin copulatory ducts and large, spherical spermathecae, but it can be easily distinguished by the distance between the atrium and epigastric furrow, which is about 2/5 the spermathecal diameter (Fig. 6) but less than 1/10 the diameter in *E.luzonica* (Logunov 2020: fig. 15).

##### Description.

**Male** (Figs 5, 6C, D, F, G). Total length 2.87. Carapace 1.64 long, 1.23 wide. Abdomen 1.28 long, 0.94 wide. Clypeus 0.08 high. Eye sizes and inter-distances: AME 0.39, ALE 0.27, PLE 0.22, AERW 1.26, PERW 1.09, EFL 0.72. Legs: I 3.73 (1.15, 1.43, 0.70, 0.45), II 2.91 (0.93, 1.00, 0.55, 0.43), III 3.41 (1.15, 1.05, 0.78, 0.43), IV 3.51 (1.10, 1.13, 0.85, 0.43). Carapace dark brown, with longitudinal, yellow area and dark radial lines on thorax, bearing dense, bilateral, white setae and sparse, golden, thin setae, denser at eye base. Fovea dark red, longitudinal. Chelicerae orange to dark brown, with two promarginal teeth and one retromarginal tooth. Endites orange to dark, white entally at tip, broadened distally. Labium almost linguiform, with several dark setae distally. Sternum dark brown, heart-shaped. Legs dark brown except middle 1/2 of metatarsi and tarsi pale or pale yellow. Abdomen suboval, dorsum rufous, dotted, with a subtrapeziform yellow patch at anterior margin, pair of transverse yellow stripes medially, two large, irregular, pale markings posteriorly; venter dark brown, with four dotted lines.

Palp (Fig. 5A–D): tibia short, about 3 times wider than long in ventral view, with tapered, straight retrolateral tibial apophysis about 1.5 times longer than tibia; cymbium about 1.8 times longer than wide in ventral view; bulb swollen, with strongly curved sperm duct; embolus forming a disc at base, followed by tapered, knife-shaped portion, coiled into about 1/4 circle, and pointed apically.

**Female** (Fig. 6A, B, E). Total length 3.15. Carapace 1.59 long, 1.18 wide. Abdomen 1.67 long, 1.28 wide. Clypeus 0.08 high. Eye sizes and inter-distances: AME 0.38, ALE 0.25, PLE 0.22, AERW 1.19, PERW 1.08, EFL 0.72. Legs: I 2.76 (0.88, 1.05, 0.48, 0.35), II 2.46 (0.75, 0.88, 0.48, 0.35), III 3.18 (1.03, 1.15, 0.65, 0.35), IV 3.21 (1.00, 1.08, 0.78, 0.35). Habitus similar to that of male except with pale yellow legs and a distinct inverted triangular, yellow area across entire surface of thorax.

Epigyne (Fig. 6A, B): atria oval, paired; copulatory openings anteriorly located, close to each other; copulatory ducts thin, strongly curved before descending posteriorly to connect with median part of ental sides of spermathecae; spermathecae almost spherical, touching; fertilization ducts lamellar, transversely extending, originating from anterior portions of spermathecae.

##### Distribution.

Known only from the type locality in Yunnan, China.

##### Comments.

Wang and Li (2021) mentioned that the generic position of *Euochinyaoi* Wang & Li, 2021 may need further confirmation. This is true for the new species and the following one as well for the same reasons.

#### ﻿ Euochin tangi sp. nov.

Taxon classificationAnimaliaAraneaeSalticidae

69D877A8-F37B-5797-82E8-B4FECABBB226

https://zoobank.org/1BEE50AF-BF19-4FA2-8292-DAA5F3923486

Fig. 7

##### Type material.

***Holotype*** ♀ (IZCAS-Ar42930), China: Yunnan: Xishuangbanna, Mengla County, Xiaolongha Village, Xishuangbanna Biodiversity Conservation Corridor, ravine rainforest (21°24.25'N, 101°36.32'E, ca 760 m alt.), 16.xi.2013, Q. Zhao and Z. Chen leg. ***Paratype*** 1♀ (IZCAS-Ar42931), same data as holotype.

##### Etymology.

The specific name is a patronym in honor of the late Guo Tang, a major collector of spiders from Xishuangbanna; noun (name) in genitive case.

##### Diagnosis.

**Figure 7.**
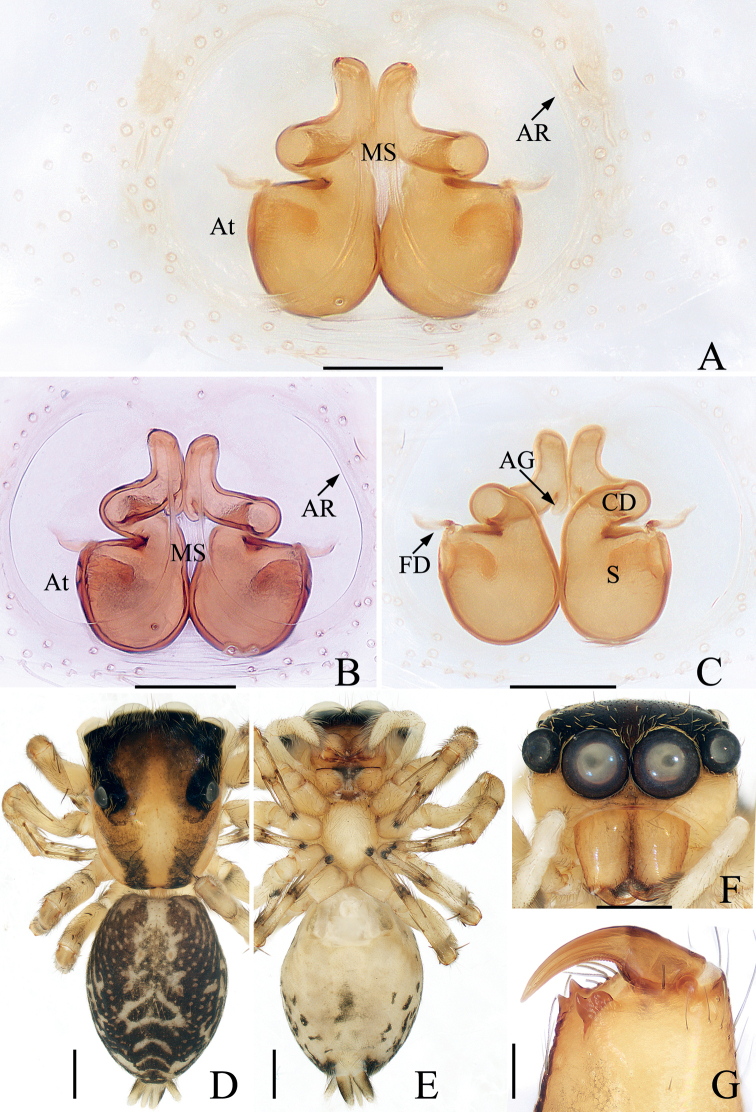
*Euochintangi* sp. nov., female holotype **A, B** epigyne, ventral **C** vulva, dorsal **D** habitus, dorsal **E** ditto, ventral **F** carapace, frontal **G** chelicera, posterior. Scale bars: 0.1 (**A–C, G**); 0.5 (**D–F**).

The new species can be easily distinguished from other congeners by the presence of anteromedial accessory glands of the copulatory ducts, and the long (longer than spermathecae) and medially fold copulatory ducts (Fig. 7A–C), which lack accessory glands of the copulatory ducts, and have short (shorter than spermathecae) and not fold copulatory ducts in others (see Metzner 2022).

##### Description.

**Female** (Fig. 7). Total length 3.82. Carapace 1.76 long, 1.41 wide. Abdomen 1.99 long, 1.48 wide. Clypeus 0.07 high. Eye sizes and inter-distances: AME 0.47, ALE 0.30, PLE 0.25, AERW 1.48, PERW 1.33, EFL 0.88. Legs: I 3.51 (1.10, 1.38, 0.63, 0.40), II 3.01 (0.95, 1.15, 0.53, 0.38), III 3.79 (1.25, 1.28, 0.83, 0.43), IV 4.07 (1.25, 1.38, 1.01, 0.43). Carapace yellow to dark brown, with pair of bilateral yellow bands and longitudinal, an irregular yellow band extending from the center of eye field to posterior margin, covered with dark and golden setae. Fovea thin, red-brown, longitudinal. Chelicerae yellow, with two promarginal teeth and one retromarginal fissidental tooth. Endites paler than chelicerae, bearing dense, dark setae at ental margins. Labium dark brown. Sternum pale to yellow, covered with brown, thin setae. Legs pale to yellow. Abdomen suboval, dorsum gray-brown to dark brown, dotted, with a longitudinal, irregular gray band anteriorly followed by two pairs of muscle depressions, then several herringbone and arched stripes, as well as several pairs of irregular gray patches laterally; venter pale yellow, with irregular dark brown patches posteromedially.

Epigyne (Fig. 7A–C): slightly wider than long; atria oval, paired; copulatory openings slit-like, beneath the anterior margin of median septum; copulatory ducts thick, posteriorly extending at origin, then curving bilaterally before folding, obliquely extending posteriorly to connect with anterior ental portions of spermathecae, with short accessory glands anteriorly; spermathecae subspherical, touching; fertilization ducts lamellar, originating from anterolateral portions of spermathecae.

**Male.** Unknown.

##### Distribution.

Known only from the type locality in Yunnan, China.

#### ﻿Genus Eupoa

Taxon classificationAnimaliaAraneaeSalticidae

Żabka, 1985

FE790FE1-4CF7-5608-8B03-03A75D2CF79C

##### Type species.

*Eupoaprima* Żabka, 1985 from Vietnam by original designation.

#### ﻿ Eupoa logunovi sp. nov.

Taxon classificationAnimaliaAraneaeSalticidae

0C671A1B-B03C-599A-82C6-14A68F59EEC1

https://zoobank.org/62C86F4D-CA74-4620-9C7A-8BCD97156B91

Figs 8 , 9

##### Type material.

***Holotype*** ♂ (IZCAS-Ar42932), China: Yunnan: Xishuangbanna, Mengla County, Xiaolongha Village, Xishuangbanna Biodiversity Conservation Corridor, ravine rainforest (21°24.25'N, 101°36.32'E, 760 ± 20 m alt.), 16.xi.2013, Q. Zhao and Z. Chen leg. ***Paratypes*** 1♂1♀ (IZCAS-Ar42933–42934), same data as holotype.

##### Etymology.

The specific name is a patronym in honor of Dmitri V. Logunov, who contributed significantly to the taxonomy of the genus *Eupoa*; noun (name) in genitive case.

##### Diagnosis.

*Eupoalogunovi* sp. nov. can be easily distinguished from other congeners by the long, twisted retrolateral femoral apophysis (longer than the tibia), which is absent or shorter than the tibia in others (see Metzner 2022). The female of this species resembles that of *E.prima* Żabka, 1985 from Vietnam in having very long copulatory ducts, but it can be easily distinguished by the presence of a concave septum (Fig. 9A) versus absent in *E.prima* (Żabka 1985: figs 167, 168), and by copulatory ducts that are connected to the baso-inner portions of spermathecae (Fig. 9B), whereas in *E.prima* they are lateral to the spermathecae (Żabka 1985: fig. 169).

##### Description.

**Figure 8.**
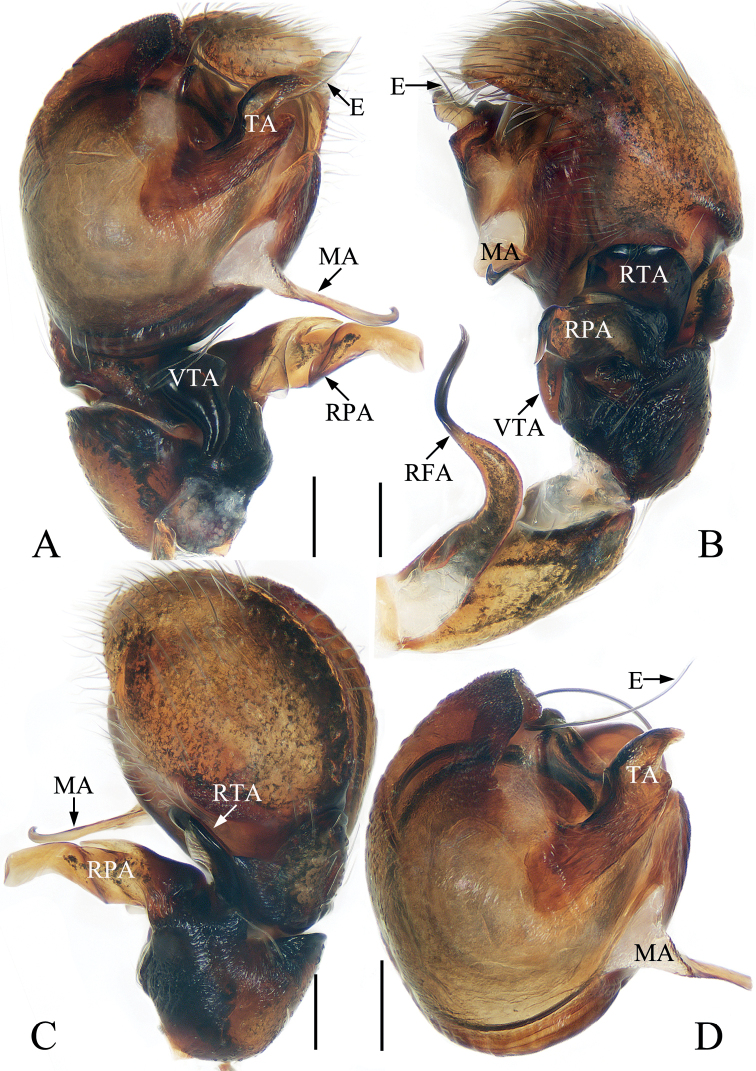
*Eupoalogunovi* sp. nov., male holotype palp **A** ventral **B** retrolateral **C** dorsal **D** bulb, ventral. Scale bars: 0.1.

**Figure 9.**
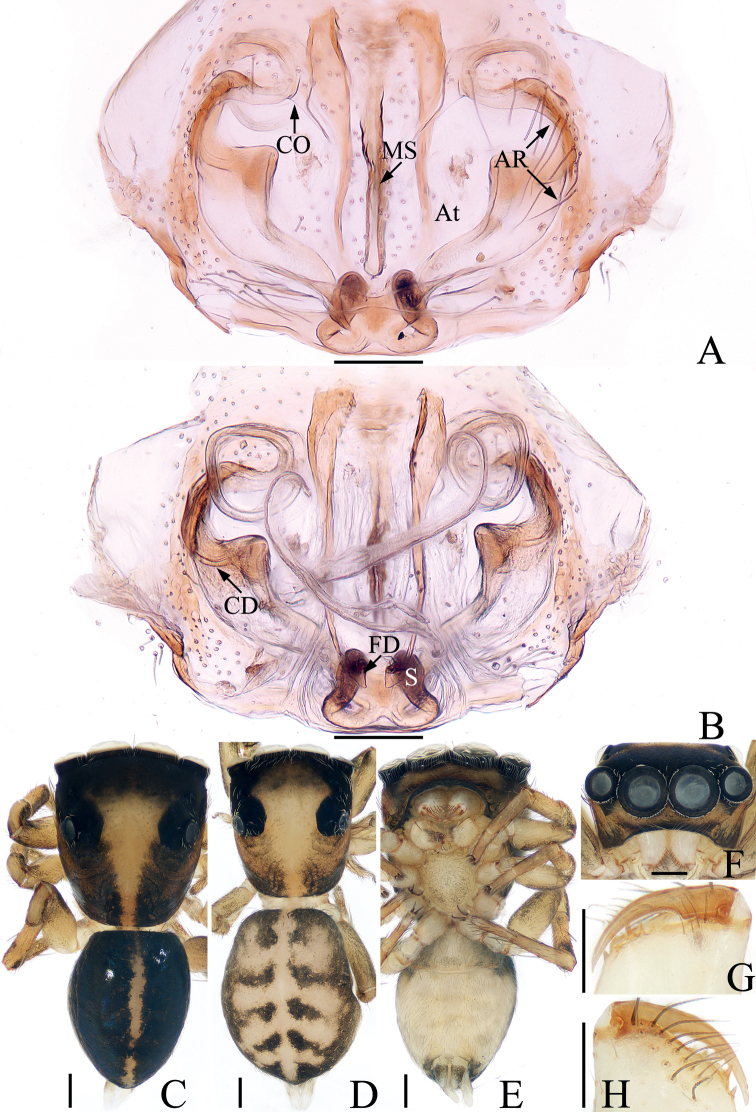
*Eupoalogunovi* sp. nov., male holotype and female paratype **A** epigyne, ventral **B** vulva, dorsal **C** male holotype habitus, dorsal **D** female paratype habitus, dorsal **E** holotype habitus, ventral **F** holotype carapace, frontal **G** holotype chelicera, posterior **H** ditto, anterior. Scale bars: 0.1 (**A, B, G, H**); 0.2 (**C–F**).

**Male** (Figs 8, 9C, E–H). Total length 2.02. Carapace 1.02 long, 0.91 wide. Abdomen 0.96 long, 0.74 wide. Clypeus 0.07 high. Eye sizes and inter-distances: AME 0.29, ALE 0.21, PLE 0.13, AERW 0.94, PERW 0.85, EFL 0.53. Legs: I 1.89 (0.60, 0.68, 0.38, 0.23), II 1.61 (0.53, 0.55, 0.30, 0.23), III 1.61 (0.50, 0.50, 0.38, 0.23), IV 2.28 (0.75, 0.80, 0.48, 0.25). Carapace yellow to dark brown, steeply sloped at posterior margin, with an inverted subtriangular yellow area extending from middle of eye field to posterior margin, bearing sparse setae at eye base. Fovea indistinct. Chelicerae pale to yellow, with two promarginal and four retromarginal teeth. Endites colored as chelicerae. Labium slightly darker than endites. Sternum almost heart-shaped, paler medially, covered with thin, brown setae. Legs yellow, with three pairs of ventral spines on metatarsi and tibiae I, respectively. Abdomen suboval, dorsum dark, somewhat mingled with blue, with longitudinal, central, narrow yellow stripe across nearly the entire surface; venter pale, setose, without markings.

Palp (Fig. 8A–D): femur about 2.5 times longer than wide in retrolateral view, with tapered, S-shaped retrolateral apophysis twisted into pointed tip; patella slightly wider than femur, with spiraled retrolateral apophysis; tibia wider than long, with strongly sclerotized, tapered ventral apophysis extending posteriorly to blunt end and squarish retrolateral apophysis; cymbium setose; bulb swollen, oval; median apophysis transversely extending in ventral view, forming small hook distally; terminal apophysis lamellar, extending antero-retrolaterally, with blunt tip; embolus filiform, coiled into circle distally, tip extending beyond cymbial tip.

**Female.** (Fig. 9A, B, D). Total length 2.20. Carapace 0.95 long, 0.82 wide. Abdomen 1.18 long, 0.94 wide. Clypeus 0.08 high. Eye sizes and inter-distances: AME 0.27, ALE 0.19, PLE 0.13, AERW 0.86, PERW 0.79, EFL 0.52. Legs: I 1.94 (0.60, 0.73, 0.38, 0.23), II 1.59 (0.48, 0.58, 0.30, 0.23), III 1.61 (0.50, 0.50, 0.38, 0.23), IV 2.46 (0.78, 0.90, 0.53, 0.25). Habitus similar to that of male except paler, with four distinct yellow bands on dorsum of abdomen.

Epigyne (Fig. 9A, B): wider than long; atrium large, suboval separated by concave septum, with pair of arched anterolateral ridges and U-shaped posterior ridge; copulatory openings anteriorly located; copulatory ducts membranous at origin, and then leading to lateral, twisted, sclerotized portions that descend obliquely and connect to the base of elongated spermathecae; fertilization ducts triangular, originating from anterior portion of spermathecae.

##### Distribution.

Known only from the type locality in Yunnan, China.

#### ﻿Genus Indomarengo

Taxon classificationAnimaliaAraneaeSalticidae

Benjamin, 2004

0B41C811-402E-5189-96EF-F6FAACBF54DA

##### Type species.

*Indomarengosarawakensis* Benjamin, 2004 from Indonesia by original designation.

#### ﻿ Indomarengo wengnan sp. nov.

Taxon classificationAnimaliaAraneaeSalticidae

E3DCC136-11CF-51C9-9BD5-3FF562BF56A7

https://zoobank.org/6FF5D3FD-D2AA-4F2F-9795-9A396EFA1F25

Fig. 10

##### Type material.

***Holotype*** ♀ (IZCAS-Ar42935), China: Yunnan: Xishuangbanna, Jinghong City, Meng’a Township, Wengnan Village, secondary forest (22°05.00'N, 100°22.22'E, 1137 ± 12 m alt.), 25.xii.2012, Q. Zhao and Z. Chen leg.

##### Etymology.

The specific name is derived from the name of the type locality and is a noun in apposition.

##### Diagnosis.

**Figure 10.**
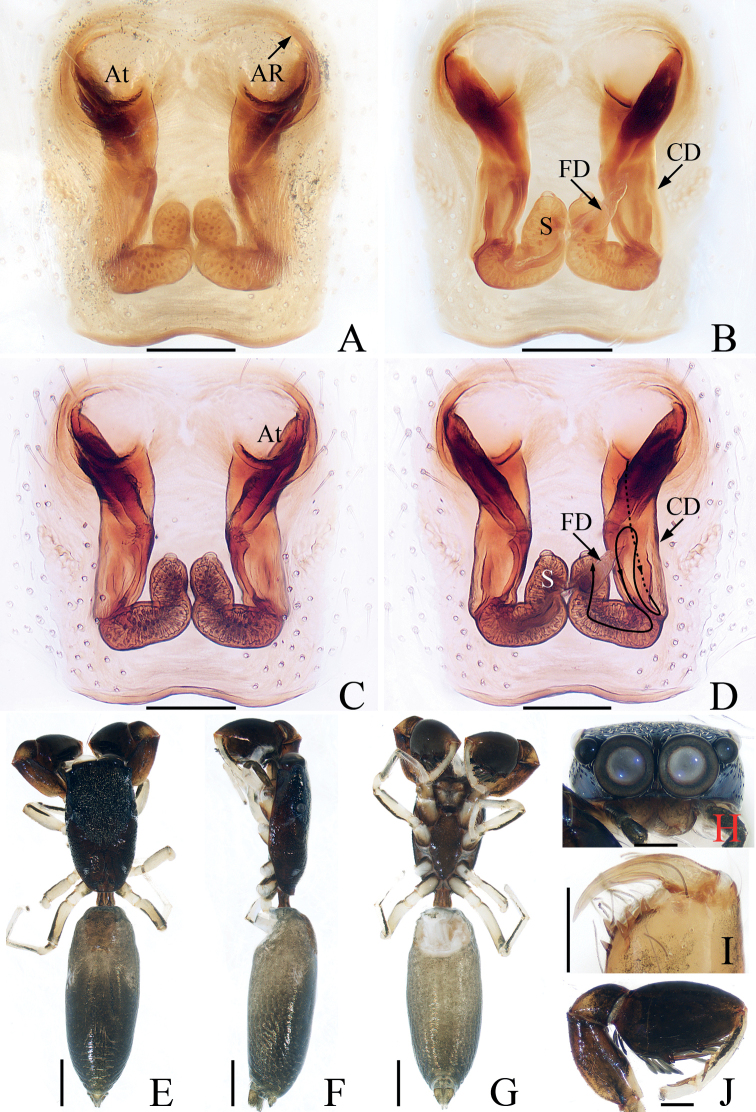
*Indomarengowengnan* sp. nov., female holotype **A, C** epigyne, ventral **B, D** vulva, dorsal **E** habitus, dorsal **F** ditto, lateral **G** ditto, ventral **H** carapace, frontal **I** chelicera, posterior **J** leg I, prolateral. Scale bars: 0.1 (**A–D, G**); 0.5 (**E–G**); 0.2 (**H, J**).

**Figure 11.**
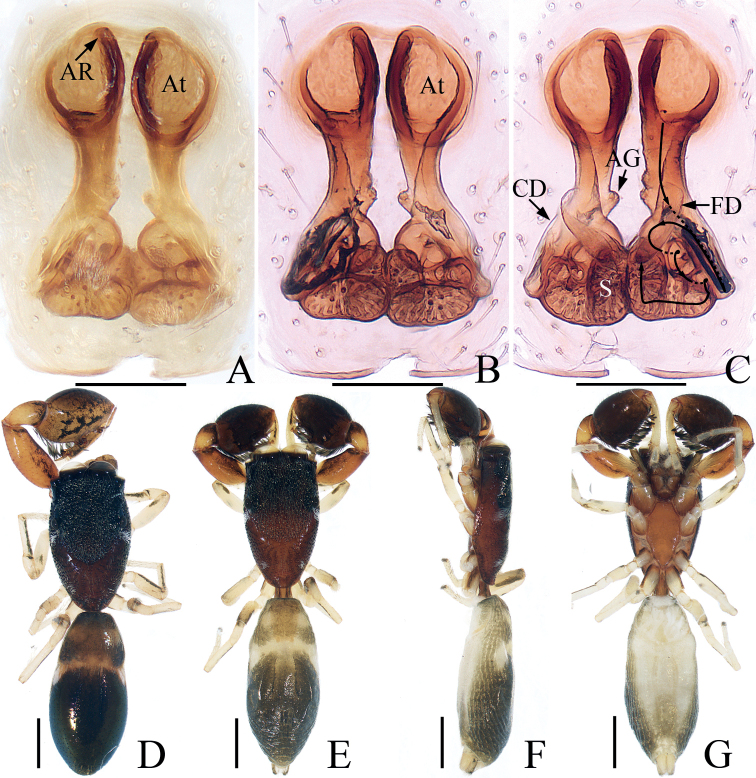
*Indomarengoyui***A, B** epigyne, ventral **C** vulva, dorsal **D** male habitus, dorsal **E** female habitus, dorsal **F** ditto, lateral **G** ditto, ventral. Scale bars: 0.1 (**A–C**); 0.5 (**D–G**).

*Indomarengowengnan* sp. nov. resembles that of *I.yui* Wang & Li, 2020 from China in having a similar habitus and L-shaped spermathecae, but it can be easily distinguished by the following: 1) atria separated from each other by more than their width (Fig. 10A–D) versus almost touching in *I.yui* (Fig. 11A–C); 2) copulatory ducts not coiled (Fig. 10A–D) versus distally coiled in *I.yui* (Fig. 11A–C). The species is also similar to *Taualaelongatus* Peng & Li, 2002 from China in the general habitus and the paired, separated atria, but it differs by the absence of accessory glands of the copulatory ducts and L-shaped spermathecae (Fig. 10A–D), whereas in *T.elongatus* the glands of the copulatory ducts are present and the spermathecae are tube-shaped (Peng and Li 2002: fig. 20).

##### Description.

**Female** (Fig. 10A–J). Total length 3.55. Carapace 1.38 long, 0.79 wide. Abdomen 2.03 long, 0.82 wide. Clypeus almost invisible. Eye sizes and inter-distances: AME 0.29, ALE 0.13, PLE 0.11, AERW 0.73, PERW 0.77, EFL 0.51. Legs: I 2.66 (0.70, 1.13, 0.63, 0.20), II 1.68 (0.50, 0.63, 0.35, 0.20), III 1.59 (0.48, 0.53, 0.38, 0.20), IV 2.23 (0.68, 0.85, 0.50, 0.20). Carapace flat, covered with thin setae anteromedially, bearing four clusters of white scales with two posterolateral to AMEs and two posterolaterally located on thorax. Chelicerae with two promarginal and three retromarginal teeth. Endites longer than wide, pale the ental sides. labium dark. Sternum elongated, almost fusiform. Legs I strongest, with enlarged tibia bearing cluster of leaf-like scales and five spines ventrally, others pale, with dark brown stripes laterally on femora and tibia. Abdomen elongated, dorsum brown to dark brown, with subtrapezoid sclerite near anterior margin and pair of indistinct white patches of setae laterally on anterior 1/3; venter gray-brown, without distinct markings.

Epigyne (Fig. 10A–D): longer than wide; atria paired, almost round, separated from each other by more than their diameter, with pair of semicircular anterolateral atrial ridges; copulatory ducts flat, broad, extending posteriorly along longitudinal axis at anterior half, before contrary extending and leading to the slender parts that slightly curved medially and connected to lateral part of spermathecae; spermathecae prominent, L-shaped, with hemispherical processes at anterior margins; fertilization ducts originating from middle of longitudinally extending portions of spermathecae.

**Male.** Unknown.

##### Distribution.

Known only from the type locality in Yunnan, China.

##### Comments.

According to the morphological characters, the new species and *I.yui* are similar to *I.thomsoni* (Wanless, 1978) and *Philateschelifer* (Simon, 1900) in having an elongated, flat body, a specific form of the copulatory ducts, and prominent spermathecae, which are absent in the type species of *Indomarengo* and *Philates* Simon, 1900, but both may not monophyletic and need further revision. We provisionally place our two species in *Indomarengo*.

#### ﻿ Indomarengo yui

Taxon classificationAnimaliaAraneaeSalticidae

Wang & Li, 2020

169D5E30-1B4E-5723-A2F7-39601DF4B5FF

Fig. 11

Indomarengo yui Wang & Li, 2020b: 51, figs 5A–D, 6A–E (male holotype, examined).

##### Material examined.

1♂1♀ (IZCAS-Ar42936–42937), China: Yunnan: Xishuangbanna, Mengla County, Huigang Village, Xilu habitat restoration area, seasonal rainforest (21°37.05'N, 101°35.27'E, 760 ± 25 m alt.), 12.xii.2012, Q. Zhao and Z. Chen leg.; 1♀ (IZCAS-Ar42938), Menglun Nature Reserve, secondary tropical forest, around garbage dump (21°54.17'N, 101°16.87'E, ca 610 m alt.), 31.xii.2018, Z. Bai et al. leg.

##### Diagnosis.

The male was thoroughly diagnosed by Wang and Li (2020b). The female resembles that of *I.thomsoni* (Wanless, 1978) from Borneo in having a similar epigyne, but it can be easily distinguished by the paired atria and L-shaped spermathecae (Fig. 11A–C), whereas there is a single atrium and irregular spermathecae in *I.thomsoni* (Wanless 1978b: fig. 8B, D, E). The species also resembles *Philateschelifer* from Indonesia, but it can be easily distinguished by having the abdomen with pair of round patches and a transverse band anteriorly (Fig. 11E), which are absent in *P.chelifer*, and by the L-shaped spermathecae (Fig. 11C), which are almost U-shaped in *P.chelifer* (Benjamin 2004: fig. 26C).

##### Description.

**Male** (Fig. 11D). See Wang and Li (2020b).

**Female** (Fig. 11A–C, E–G). Total length 3.20. Carapace 1.20 long, 0.79 wide. Abdomen 1.73 long, 0.76 wide. Clypeus almost invisible. Eye sizes and inter-distances: AME 0.28, ALE 0.12, PLE 0.11, AERW 0.73, PERW 0.78, EFL 0.51. Legs: I 2.48 (0.68, 1.05, 0.55, 0.20), II 1.57 (0.48, 0.58, 0.33, 0.18), III 1.51 (0.48, 0.50, 0.35, 0.18), IV 2.07 (0.64, 0.80, 0.45, 0.18). Carapace flat, red-brown to dark, covered with thin setae, bearing four clusters of white scales. Chelicerae, endites, labium, sternum, and legs similar to that of male. Abdomen elongated, dorsum brown to dark brown, with subtrapezoid sclerite, pair of round pale patches near anterior margin, followed by transverse pale band bearing pair of white patches at lateral margins; venter pale.

Epigyne (Fig. 11A–C): longer than wide, with arched atrial ridge anteriorly; atria paired, oval, nearly touching; copulatory openings located at base of atria; copulatory ducts posterolaterally extending before returning to the middle part, then continuing, coiled into two semicircles, connecting to the lateral sides of spermathecae; spermathecae prominent, almost L-shaped, with small, hemispheric processes at anterior margins; fertilization ducts originating from anterior portions of longitudinal extensions of spermathecae.

##### Distribution.

Known only from the type locality in Yunnan, China.

#### ﻿Genus Laufeia

Taxon classificationAnimaliaAraneaeSalticidae

Simon, 1889

4F43BAF1-D98E-5DDC-9EBC-E127A62EBC58

##### Type species.

*Laufeiaaenea* Simon, 1889 from Japan by original designation.

#### ﻿ Laufeia zhangae sp. nov.

Taxon classificationAnimaliaAraneaeSalticidae

E2DEE22C-0E3E-53BA-A401-A2F12D537CAD

https://zoobank.org/27C8FE88-B067-4B7B-A496-E9B377518ECA

Figs 12 , 13

Laufeia squamata Logunov & Jäger, 2015: 355, figs 33–36 (♂, mismatched).

##### Type material.

***Holotype*** ♂ (IZCAS-Ar42939), China: Yunnan: Xishuangbanna, Jihong City, Mengyang Township, seasonal forest (22°09.77'N, 100°52.55'E, 860 ± 30 m alt.), 22.xii.2012, Q. Zhao and Z. Chen leg. ***Paratypes*** 1♂ (IZCAS-Ar42940), Menglun Township, 55 km from Xishuangbanna National Nature Reserve, artificial *Ficusmicrocarpa* forest (21°54.97'N, 100°16.05'E, ca 610 m alt.), 21.viii.2011, Q. Zhao leg.; 1♀ (IZCAS-Ar42941), 55 km from Xishuangbanna National Nature Reserve, seasonal rainforest (21°57.70'N, 101°12.52'E, 670 ± 30 m alt.), Q. Zhao and Z. Chen leg.; 1♀ (IZCAS-Ar42942), Mengyang Township, seasonal rainforest off Baihuashan tunnel (22°09.53'N, 101°55.21'E, 860 ± 12 m alt.), 16. xii.2012, Q. Zhao and Z. Chen leg.

##### Etymology.

The species name is a patronym in honor of Ms Junxia Zhang, who has contributed greatly to the taxonomy of jumping spiders worldwide; noun (name) in genitive case.

##### Diagnosis.

**Figure 12.**
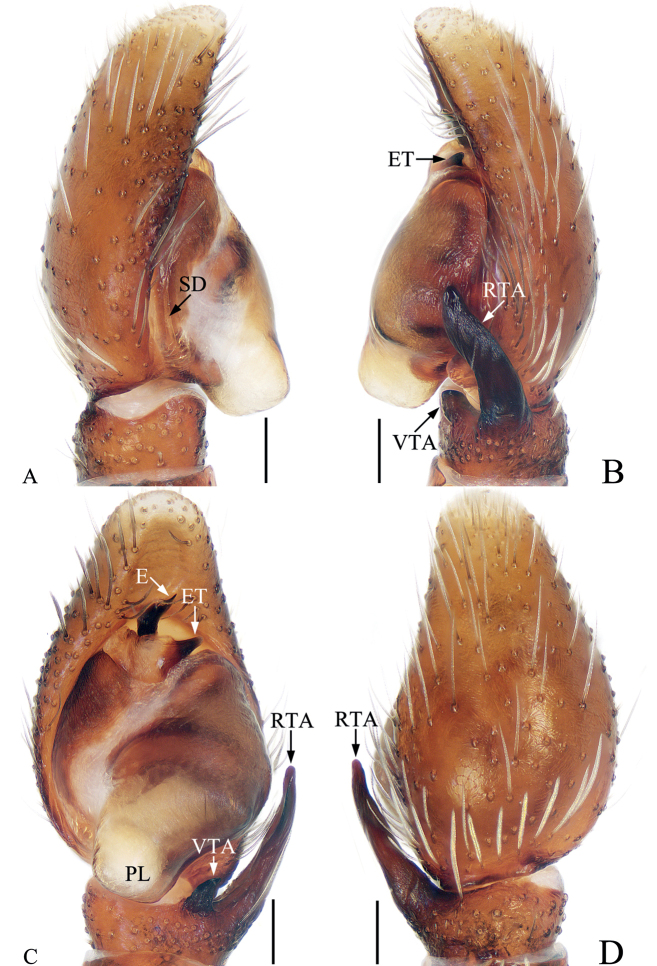
*Laufeiazhangae* sp. nov., male holotype palp **A** prolateral **B** retrolateral **C** ventral **D** dorsal. Scale bars: 0.1.

**Figure 13.**
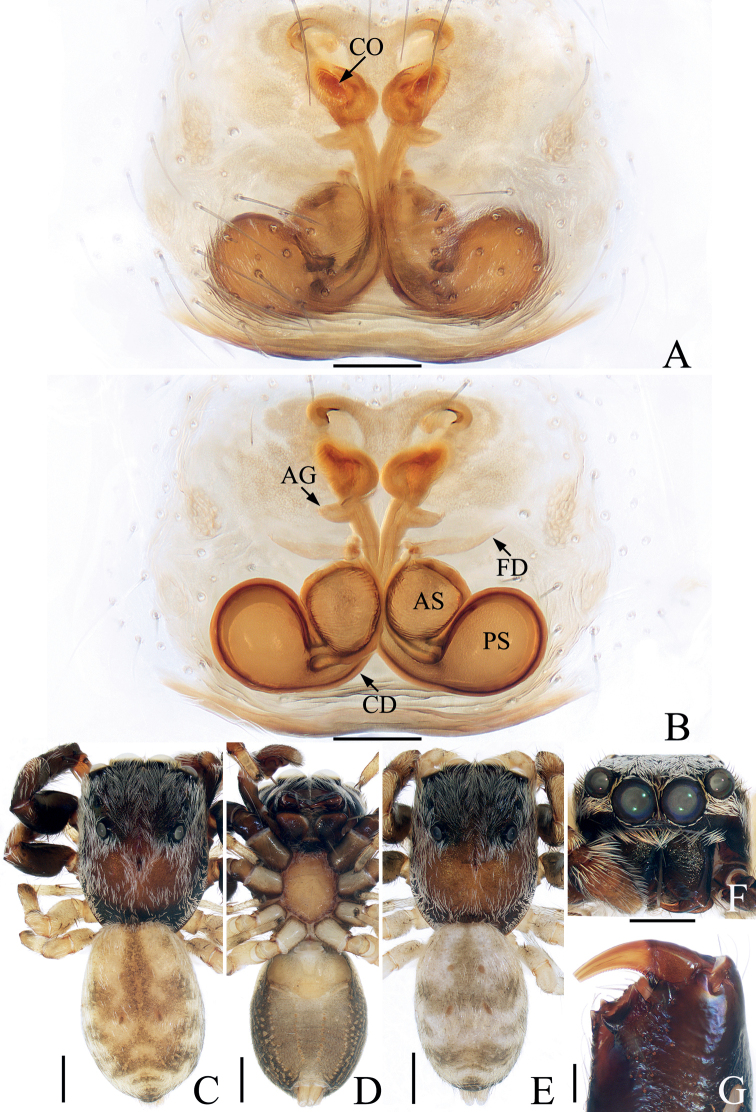
*Laufeiazhangae* sp. nov., male holotype and female paratype **A** epigyne, ventral **B** vulva, dorsal **C** male holotype habitus, dorsal **D** ditto, ventral **E** female paratype habitus, dorsal **F** holotype carapace, frontal **G** holotype chelicera, posterior. Scale bars: 0.1 (**A, B, G**); 0.5 (**C–F**).

*Laufeiazhangae* sp. nov. closely resembles *L.aenea* Simon, 1889 from China, Korea, and Japan in general habitus and copulatory organs, but it can be easily distinguished by the following: 1) embolus lacks a branched projection (Fig. 12C) versus branched projection present in *L.aenea* (Ikeda 1998: fig. 6); 2) tibia with a subtriangular ventral apophysis (Fig. 12C) versus ventral apophysis indistinct in *L.aenea* (Ikeda 1998: fig. 6); 3) copulatory openings slit-like (Fig. 13A) versus openings oval in *L.aenea* (Ikeda 1998: fig. 7); 4) copulatory ducts with proximal conical accessory glands (Fig. 13B) versus glands indistinct in *L.aenea* (Ikeda 1998: fig. 8).

##### Description.

**Male** (Figs 12, 13C, D, F, G). Total length 3.56. Carapace 1.83 long, 1.39 wide. Abdomen 1.92 long, 1.31 wide. Clypeus 0.03 high. Eye sizes and inter-distances: AME 0.36, ALE 0.25, PLE 0.22, AERW 1.18, PERW 0.88, EFL 0.78. Legs: I 3.61 (1.13, 1.50, 0.58, 0.40), II 2.96 (0.90, 1.13, 0.53, 0.40), III 2.96 (0.95, 1.08, 0.53, 0.40), IV 3.01 (0.90, 1.13, 0.58, 0.40). Carapace squarish, red-brown to dark brown, covered with dense, white setae, and golden setae on anterior eye bases and clypeus. Fovea dark, longitudinal. Chelicerae dark red to dark, with two promarginal teeth and one retromarginal fissidental tooth with two cusps. Endites red-brown to dark, broadened distally, bearing dense, dark setae at ental margins. Labium dark, almost linguiform. Sternum yellow, covered with pale, thin setae. Legs pale yellow to dark brown. Abdomen suboval, with large, irregular, dark brown patch and two pairs of muscle depressions, covered with short, white, thin setae; venter brown to dark brown, dotted laterally, with pair of dotted lines medially.

Palp (Fig. 12A–D): tibia short, about 1.5 times wider than long in retrolateral view, with strongly sclerotized, subtriangular ventral apophysis pointed apically, and tapered retrolateral apophysis slightly curved, extending antero-prolaterally to a blunt tip in retrolateral view; cymbium about 1.7 times longer than wide in ventral view, with sparse, long, white scales at dorsum of proximal portion; bulb longer than wide, with subtrapezoid posterior lobe; embolus strongly sclerotized, short, curved towards retrolateral side distally, with a pointed tip directed towards about 1:00 position, and a tapered, blunt basal tooth.

**Female** (Fig. 13A, B, E). Total length 3.15. Carapace 1.67 long, 1.26 wide. Abdomen 1.64 long, 1.13 wide. Clypeus 0.03 high. Eye sizes and inter-distances: AME 0.33, ALE 0.23, PLE 0.19, AERW 1.05, PERW 0.87, EFL 0.69. Legs: I 2.66 (0.83, 1.20, 0.38, 0.25), II 2.36 (0.78, 0.95, 0.38, 0.25), III 2.60 (0.85, 0.95, 0.50, 0.30), IV 2.89 (0.88, 1.10, 0.58, 0.33). Habitus similar to that of male except paler.

Epigyne (Fig. 13A, B): wider than long, with pair of shallow hoods anteriorly; copulatory openings anteriorly located, slit-like; copulatory ducts swollen at origin, extending posteriorly to connect with the base of ental sides of posterior chambers of spermathecae, with proximal, conical accessory glands; spermathecae divided into two sub-spherical chambers; fertilization ducts anterior to anterior chamber of spermathecae, extended transversely.

##### Distribution.

China (Yunnan), Vietnam.

#### ﻿Genus Rhene

Taxon classificationAnimaliaAraneaeSalticidae

Thorell, 1869

485E26F3-FD93-5F81-A88F-AEBEFABF25DA

##### Type species.

*Rhanisflavigera* C. L. Koch, 1846 from Indonesia by original designation.

#### ﻿ Rhene triapophyses

Taxon classificationAnimaliaAraneaeSalticidae

Peng, 1995

380B4182-06B4-5B2F-BB2F-833BEFD47B61

Figs 14 , 15

Rhene triapophyses Peng, 1995: 35, figs 1–5 (male holotype, not examined); Peng 2020: 395, fig. 288a–e.

##### Material examined.

1♂3♀ (IZCAS-Ar42943–42946), China: Yunnan, Xishuangbanna, Mengla County, Menglun Town, Xishuangbanna Tropical Botanical Garden, Yulinjiegou (21°55.05'N, 101°16.24'E, ca 570 m alt.), 19.xii.2018, X. Mi et al. leg.; 1♂1♀ (IZCAS-Ar42947–42948), 1 site in Mafengzhai (21°53.45'N, 101°17.40'E, ca 543 m alt.), 29.ix.2019, Y. Tong et al. leg.

##### Diagnosis.

**Figure 14.**
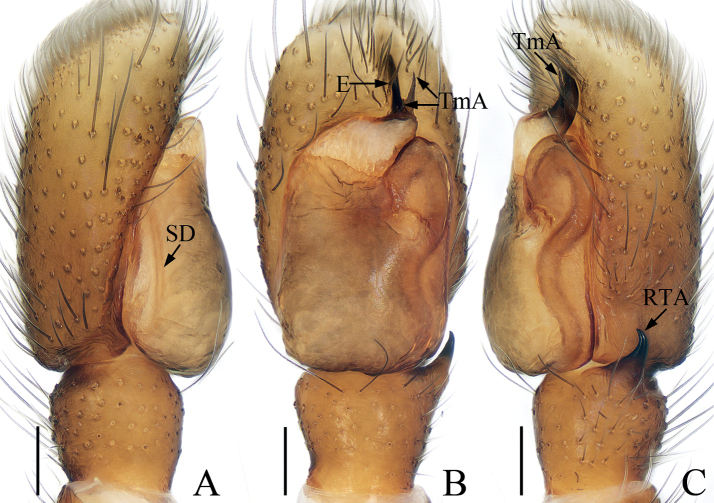
*Rhenetriapophyses*, male palp **A** prolateral **B** ventral **C** retrolateral. Scale bars: 0.1.

**Figure 15.**
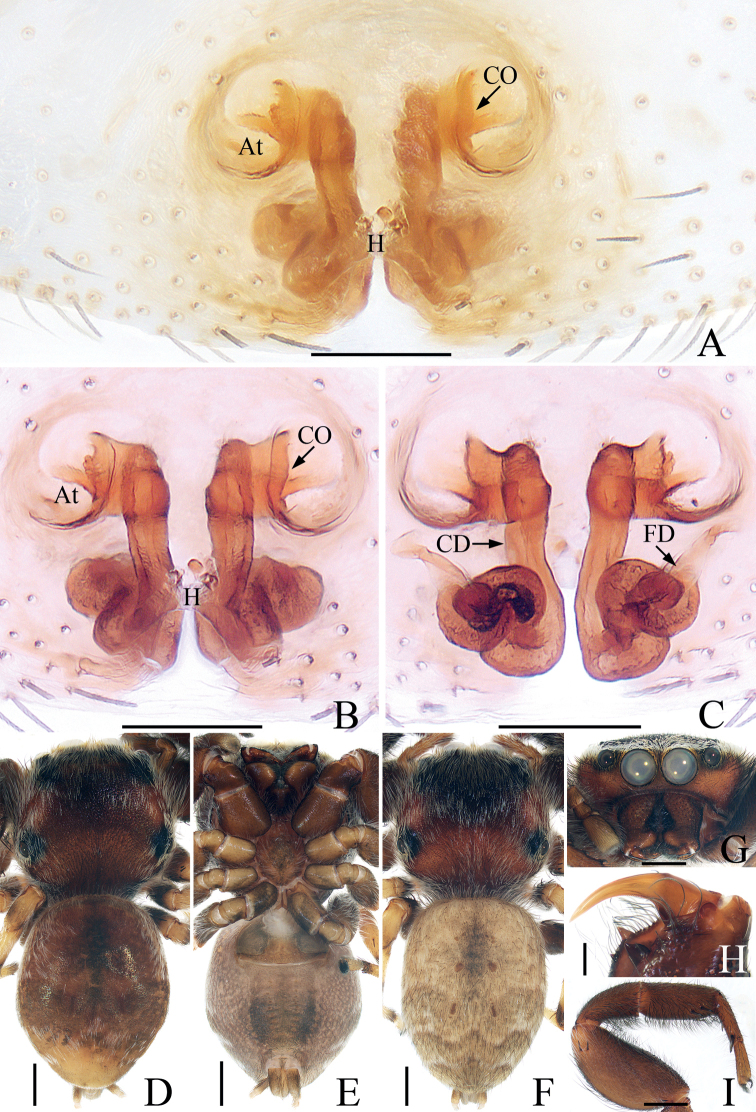
*Rhenetriapophyses***A, B** epigyne, ventral **C** vulva, dorsal **D** male habitus, dorsal **E** ditto, ventral **F** female habitus, dorsal **G** male carapace, frontal **H** male chelicera, posterior **I** male leg I, prolateral. Scale bars: 0.1 (**A–C, H**); 0.5 (**D–F, I**); 0.2 (**G**).

*Rhenetriapophyses* Peng, 1995 closely resembles that of *R.setipes* Żabka, 1985 from China, Vietnam and Japan in the general shape of the habitus and copulatory organs, but it differs in the following: 1) embolic division includes two terminal apophyses (Fig. 14B) versus only one terminal apophysis in *R.setipes* (Żabka 1985: fig. 563); 2) anteromedially, retrolateral tibial apophysis extending antero-retrolaterally (Fig. 14C) versus extending antero-prolaterally in *R.setipes* (Żabka 1985: fig. 564); 3) female almost indistinguishable from *R.setipes* except in the form of the copulatory ducts and atria (Fig. 15A–C vs Tanikawa 1993: figs 10, 11).

##### Description.

**Male** (Figs 14, 15D, E, G–I). Total length 4.07. Carapace 1.93 long, 1.90 wide. Abdomen 2.30 long, 1.83 wide. Clypeus 0.08 high. Eye sizes and inter-distances: AME 0.42, ALE 0.22, PLE 0.20, AERW 1.43, PERW 1.87, EFL 1.24. Legs: I 4.66 (1.63, 1.85, 0.68, 0.50), II 3.19 (1.08, 1.13, 0.58, 0.40), III 2.98 (1.00, 0.98, 0.60, 0.40), IV 3.88 (1.33, 1.40, 0.75, 0.40). Carapace red-brown to dark brown, almost hexagonal, with large, irregular dark brown patch at center of eye field, covered with dense setae. Fovea indistinct. Chelicerae red-brown, with two promarginal teeth and one retromarginal tooth, the paturon covered by papillae, with distinct incision on anterior surface. Endites typical, bearing dark setae entally. Labium darker than endites. Sternum almost oval, bearing pale, thin setae. Legs I strongest, with enlarged femora, two pairs of ventral spines on tibia and three ventral spines on metatarsi, other legs yellow to dark brown, with slightly enlarged femora. Abdomen suboval, dorsum red-brown, yellow posteriorly, with an irregular, longitudinal dark patch anteromedially followed by a broad, transverse, dark brown band, covered with short, pale white, thin setae and wholly covered by large scutum; venter brown, with a pair of longitudinal, dark stripes medially.

Palp (Fig. 14A–D): tibia almost as long as wide, with tapered retrolateral apophysis distally curved inward to a pointed tip; cymbium about 1.5 times longer than wide; bulb slightly swollen posteromedially, with tapered sperm duct, sinuous retrolaterally; embolus originating from middle of anterior margin of bulb, bar-shaped, blunt apically, division with two spiny apophyses.

**Female** (Fig. 15A–C, F). Total length 4.32. Carapace 2.04 long, 1.96 wide. Abdomen 2.50 long, 1.86 wide. Clypeus 0.08 high. Eye sizes and inter-distances: AME 0.40, ALE 0.22, PLE 0.20, AERW 1.43, PERW 1.96, EFL 1.29. Legs: I 4.00 (1.45, 1.55, 0.55, 0.45), II 3.13 (1.08, 1.15, 0.50, 0.40), III 3.08 (1.03, 1.05, 0.60, 0.40), IV 4.09 (1.33, 1.53, 0.83, 0.40). Habitus similar to that of male except paler and without dorsal scutum on abdomen.

Epigyne (Fig. 15A–C): wider than long, with broad posterior hood distant from epigastric furrow; atria paired, oval, separated from each other by slightly more than width of epigynal hood; copulatory ducts long, transversely extending before curving 90° then descending posteriorly, continuing into an S-shaped coil; spermathecae indistinct; fertilization ducts lamellar, extending anterolaterally.

##### Distribution.

China (Yunnan).

##### Comments.

The male of the new material is almost identical with the holotype in palpal and cheliceral structure except detail difference in the length of the apophyses of embolic division. Moreover, material studied in this paper were collected from the same locality as holotype in Menglun County, Xishuangbanna, China.

#### ﻿Genus Simaetha

Taxon classificationAnimaliaAraneaeSalticidae

Thorell, 1881

92F16019-DB39-5233-A526-BF357818A4AB

##### Type species.

*Simaethathoracica* Thorell, 1881 from Australia by original designation.

#### ﻿ Simaetha huigang sp. nov.

Taxon classificationAnimaliaAraneaeSalticidae

0D52B787-B92A-5F40-81AE-9989109AF81B

https://zoobank.org/C45C3830-6985-49C1-B781-9FF7B7EFB63C

Figs 16 , 17

##### Type material.

***Holotype*** ♂ (IZCAS-Ar42949), China: Yunnan: Xishuangbanna, Mengla County, Huigang Village, Xilu habitat restoration area, seasonal rainforest (21°37.05'N, 101°35.27'E, 764 ± 25 m alt.), 12.xii.2012, Q. Zhao and Z. Chen leg. ***Paratypes*** 1♀ (IZCAS-Ar42950), same data as holotype; 1♂ (IZCAS-Ar42951), Menglun Town, Menglun Nature Reserve, 2 site of Leprosy Village (21°53.59'N, 101°17.30'E, ca 550 m alt.), 4.v.2019, Y. Tong et al. leg; 1♂1♀ (IZCAS-Ar42952–42953), Xiaolongha Village, diversity conservation corridor of Xishuangbanna National Nature Reserve, seasonal rainforest (21°24.19'N, 101°37.03'E, 657 ± 15 m alt.), 29.xi.2012, Q. Zhao and Z. Chen leg.

##### Etymology.

The species name is a noun in apposition derived from the holotype locality.

##### Diagnosis.

**Figure 16.**
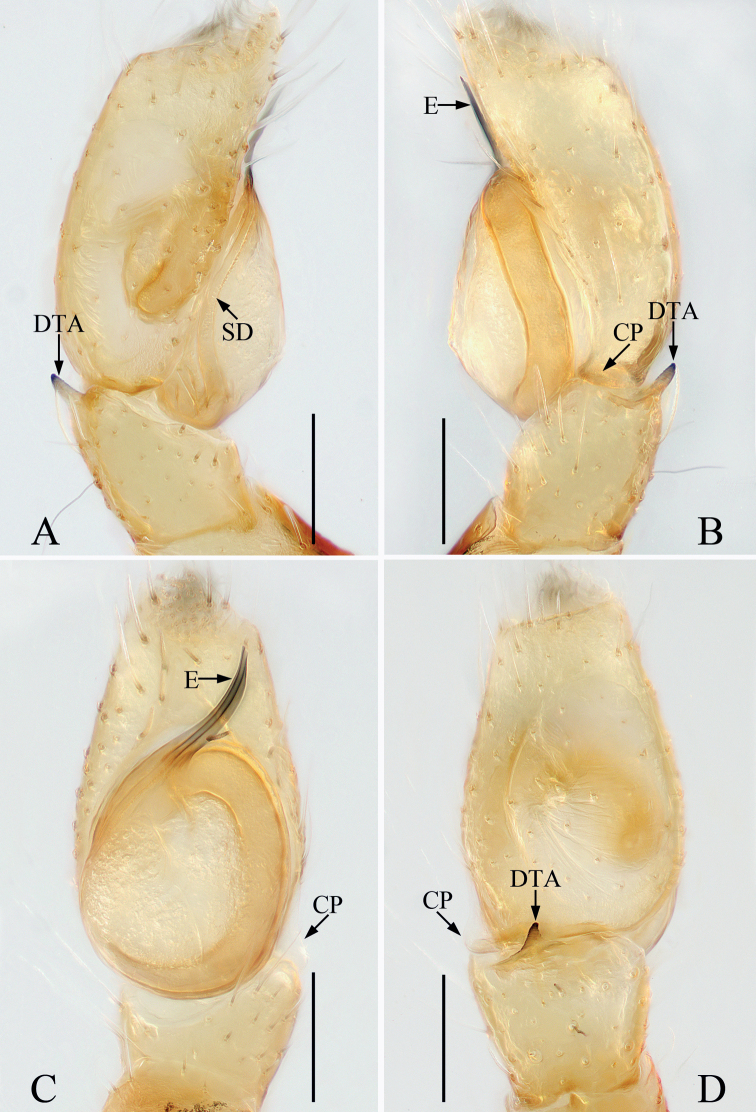
*Simaethahuigang* sp. nov., male holotype palp **A** prolateral **B** retrolateral **C** ventral **D** dorsal. Scale bars: 0.1.

**Figure 17.**
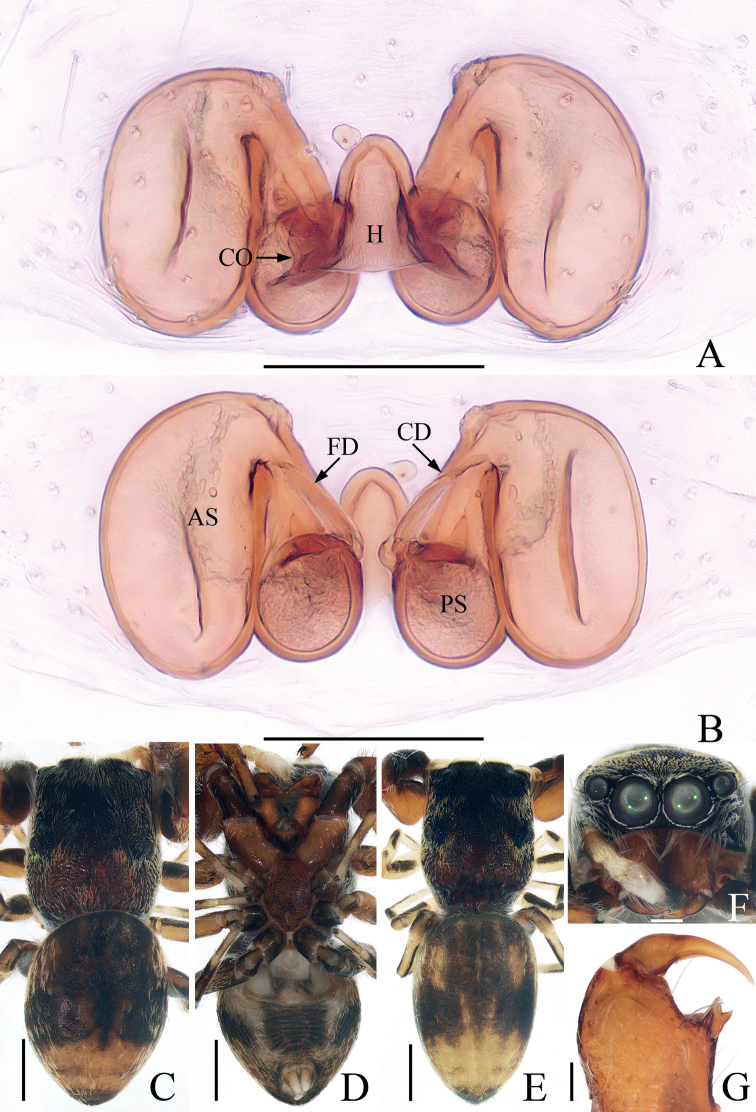
*Simaethahuigang* sp. nov., male holotype and female paratype **A** epigyne, ventral **B** vulva, dorsal **C** male holotype habitus, dorsal **D** ditto, ventral **E** female paratype habitus, dorsal **F** holotype carapace, frontal **G** holotype chelicera, anterior. Scale bars: 0.1 (**A, B, G**); 0.5 (**C–E**); 0.2 (**F**).

*Simaethahuigang* sp. nov. closely resembles *S.cheni* from China in the general shape of the habitus and copulatory organs, but it differs in the following: 1) dorsal tibial apophysis less than 1/4 tibial length in retrolateral view (Fig. 16B) versus more than 1/2 tibial length in *S.cheni* (Wang and Li 2021: fig. 18C); 2) cheliceral paturon lacks process (Fig. 17F, G) versus process present mediolaterally on anterior surface in *S.cheni* (Wang and Li 2021: fig. 19G, H); 3) epigynal hood almost as long as posterior chamber of spermathecae (Fig. 17A, B) versus less than 1/2 length of posterior chamber of spermathecae in *S.cheni* (Wang and Li 2021: fig. 19A–C).

##### Description.

**Male** (Figs 16, 17C, D, F, G). Total length 2.77. Carapace 1.36 long, 1.11 wide. Abdomen 1.60 long, 1.17 wide. Clypeus 0.02 high. Eye sizes and inter-distances: AME 0.32, ALE 0.17, PLE 0.15, AERW 0.92, PERW 1.04, EFL 0.75. Legs: I 2.96 (1.00, 1.20, 0.43, 0.33), II 2.11 (0.68, 0.73, 0.40, 0.30), III 1.93 (0.65, 0.60, 0.40, 0.28), IV 2.41 (0.85, 0.83, 0.43, 0.30). Carapace red-brown to dark, squarish, slightly narrowed at anterior half, covered with dense setae and scales. Fovea indistinct. Chelicerae yellow-brown, with two promarginal and one retromarginal fissidental tooth with two cusps. Endites longer than wide, bearing dense setae at ental margins. Labium darker than endites. Sternum 1.5 times longer than wide, covered by pale, long, thin setae. Legs I strongest, covered with pale and blue scales on enlarged femora and tibiae, with two pairs of spines on tibiae and metatarsi, respectively; other legs yellow to dark. Abdomen oval, the dorsum with a large, irregular dark band followed by a broad, transverse, yellow band, entirely covered by large scutum, bearing short, pale, thin setae; venter dark brown, laterally with pair of longitudinal, pale setal stripes.

Palp (Fig. 16A–D): tibia wider than long in ventral view, with short, straight, lamellar dorsal apophysis slightly pointed apically; cymbium about 1.8 times longer than wide, with lamellar, proximal retrolateral process; bulb almost round, with sperm duct extending along submargin; embolus flat, about 1/2 the bulb length, originating from antero-prolateral portion of bulb, slightly curved medially, blunt apically.

**Female** (Fig. 17A, B, E). Total length 2.99. Carapace 1.32 long, 1.02 wide. Abdomen 1.70 long, 1.09 wide. Clypeus 0.02 high. Eye sizes and inter-distances: AME 0.30, ALE 0.16, PLE 0.15, AERW 0.91, PERW 1.02, EFL 0.67. Legs: I 2.19 (0.73, 0.73, 0.43, 0.30), II 1.70 (0.55, 0.60, 0.30, 0.25), III 1.64 (0.53, 0.53, 0.33, 0.25), IV 2.23 (0.78, 0.80, 0.40, 0.25). Habitus similar to that of male except paler.

Epigyne (Fig. 17A, B): wider than long, with large, central, bell-shaped hood almost equal in length to posterior chamber of spermathecae; copulatory openings lateral to base of hood, slit-like; copulatory ducts thick, connected with anterior portions of anterior chambers of spermathecae; spermathecae divided into two chambers, anterior chamber oval, extending posteriorly, posterior chamber almost spherical, separated from each other by 1/4 their diameter; fertilization ducts originating from anterior portions of posterior chamber of spermathecae, extending anterolaterally.

##### Distribution.

Known only from the type locality in Yunnan, China.

#### ﻿Genus Synagelides

Taxon classificationAnimaliaAraneaeSalticidae

Strand, 1906

65474F3B-E361-5458-9F2B-05571CFBE8C9

##### Type species.

*Synagelidesagoriformis* Strand, 1906 from Japan by original designation.

#### ﻿ Synagelides cheni sp. nov.

Taxon classificationAnimaliaAraneaeSalticidae

78C802F2-AA16-5C75-B943-B7683919E693

https://zoobank.org/D1842C11-3FDF-44AC-AA58-271BEBAF31F2

Fig. 18

##### Type material.

***Holotype*** ♀ (IZCAS-Ar42954), China: Yunnan: Xishuangbanna, Mengla County, Menglun Township, 55 kilometers from Xishuangbanna National Nature Reserve, ravine rainforest (21°57.68'N, 101°12.03'E, 718 ± 11 m alt.), 12.xi.2013, Q. Zhao and Z. Chen leg. ***Paratype*** 1♀ (IZCAS-Ar42955), same data as holotype.

##### Etymology.

The specific name is a patronym in honor of Zhigang Chen, one of the collectors of the new species; noun (name) in genitive case.

##### Diagnosis.

**Figure 18.**
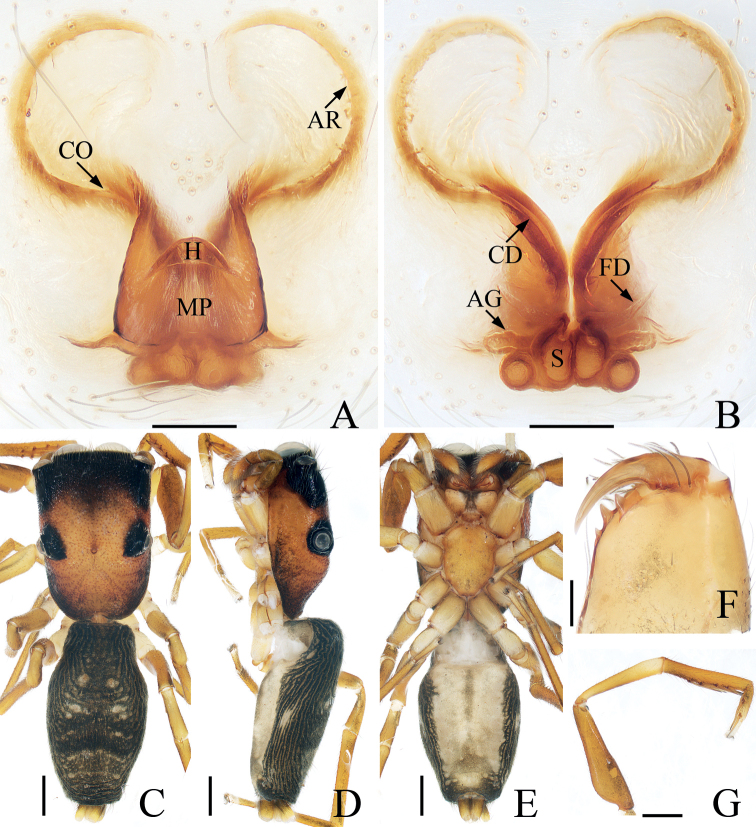
*Synagelidescheni* sp. nov., female holotype **A** epigyne, ventral **B** vulva, dorsal **C** habitus, dorsal **D** ditto, lateral **E** ditto, ventral **F** chelicera, posterior **G** leg I, prolateral. Scale bars: 0.1 (**A, B, F**); 0.5 (**C–E, G**).

*Synagelidescheni* sp. nov. resembles that of *S.tangi* Liu, Chen, Xu & Peng, 2017 from China in having anteriorly located, paired, arched atrial ridges and a centrally located epigynal hood, but it can be easily distinguished by the following: 1) atrial ridges occupying nearly entire anterior 1/2 of epigyne (Fig. 18 A, B) versus occupying about anterior 1/3 of the epigyne in *S.tangi* (Liu et al. 2017: figs 4C, D, 5E, F); 2) epigynal hood about three times wider than long (Fig. 18A) versus about as long as wide in *S.tangi* (Liu et al. 2017: figs 4C, 5E).

##### Description.

**Female** (Fig. 18). Total length 4.38. Carapace 2.04 long, 1.46 wide. Abdomen 2.33 long, 1.28 wide. Clypeus 0.05 high. Eye sizes and inter-distances: AME 0.50, ALE 0.31, PLE 0.29, AERW 1.48, PERW 1.45, EFL 1.14. Legs: I 3.74 (1.58, 1.28, 0.55, 0.33), II 3.56 (1.10, 1.33, 0.75, 0.38), III 3.71 (1.10, 1.30, 0.93, 0.38), IV 5.13 (1.45, 1.98, 1.25, 0.45). Carapace stippled, yellow to dark, covered with dark and pale setae. Fovea oval, hollow. Chelicerae yellow, with two promarginal teeth and one retromarginal fissidental tooth with two cusps. Endites slightly paler than chelicerae. Labium dark yellow, bearing several dark setae at distally. Sternum yellow, almost shield-like. Legs pale to yellow, with four and two pairs of ventral spines on metatarsi and tibiae I, respectively. Abdomen elongated, dorsum dark brown, with two pairs of muscle depressions medially, two wavy, transverse dark stripes and two transverse dotted lines posteriorly; venter pale, with longitudinal, gray-brown stripe anteromedially.

Epigyne (Fig. 18A, B): slightly longer than wide, with pair of arched atrial ridges occupying nearly entire anterior half, subtrapezoid median plate bearing an inverted boat-shaped hood about three times wider than long; copulatory openings small, situated at base of atrial ridges; copulatory ducts descending, forming C-shape, terminally with transversely extending, bar-shaped accessory glands; spermathecae almost L-shaped, with spherical lateral and oval ental portions; fertilization ducts lamellar, originating from anterior margins of ental portions of spermathecae, extending transversely.

**Male.** Unknown.

##### Distribution.

Known only from the type locality in Yunnan, China.

## Supplementary Material

XML Treatment for Bocusoides

XML Treatment for Bocusoides zhaoi

XML Treatment for Chalcovietnamicus

XML Treatment for Chalcovietnamicus lii

XML Treatment for Euochin

XML Treatment for Euochin mii

XML Treatment for Euochin tangi

XML Treatment for Eupoa

XML Treatment for Eupoa logunovi

XML Treatment for Indomarengo

XML Treatment for Indomarengo wengnan

XML Treatment for Indomarengo yui

XML Treatment for Laufeia

XML Treatment for Laufeia zhangae

XML Treatment for Rhene

XML Treatment for Rhene triapophyses

XML Treatment for Simaetha

XML Treatment for Simaetha huigang

XML Treatment for Synagelides

XML Treatment for Synagelides cheni
